# The Effect of Intensive Blood Pressure Reduction on Perihematomal Edema After Intracerebral Hemorrhage: A Systematic Review and Meta‐Analysis

**DOI:** 10.1002/brb3.71724

**Published:** 2026-09-14

**Authors:** Xianxian Li, Chengguang Zou, Yuchao Jia, Chensheng Pan, Xiaodong Ye, Suiqiang Zhu, Shanshan Huang

**Affiliations:** ^1^ Department of Neurology Tongji Hospital, Tongji Medical College, Huazhong University of Science and Technology Wuhan Hubei China

**Keywords:** blood pressure, brain edema, cerebral hemorrhage, meta‐analysis

## Abstract

**Background:**

Perihematomal edema (PHE) is a radiological marker of intracerebral hemorrhage (ICH)‐induced secondary brain injury. Whether intensive blood pressure (BP) reduction attenuates PHE expansion remains uncertain. We conducted a systematic review and meta‐analysis to assess the effect of intensive BP reduction on approximately 24‐h PHE expansion in patients with spontaneous ICH.

**Methods:**

MEDLINE, Embase, and the Cochrane Library were searched from database inception to January 2026. Eligible studies compared intensive versus standard BP reduction in patients with spontaneous ICH and reported PHE‐related outcomes. Risk of bias was assessed using the Cochrane risk‐of‐bias tool. Pooled estimates were calculated using the inverse‐variance method with a fixed‐effect model.

**Results:**

Six studies were included in the qualitative synthesis, and 4 studies were included in the quantitative meta‐analysis, comprising 1061 patients in the intensive BP reduction group and 966 patients in the standard BP management group. Intensive BP reduction was associated with smaller approximately 24‐h PHE expansion than standard BP management (mean difference, −0.50 mL; 95% CI, −0.89 to −0.12; *p* = 0.010). No statistical heterogeneity was observed (*I* ^2^ = 0%).

**Conclusions:**

Intensive BP reduction was associated with smaller approximately 24‐h PHE expansion in patients with spontaneous ICH. Further standardized studies are needed to confirm the clinical relevance of this effect.

## Graphical abstract

This systematic review and meta‐analysis suggests that intensive blood pressure reduction is associated with modestly smaller approximately 24‐h perihematomal edema expansion after spontaneous intracerebral hemorrhage, supporting further standardized trials to clarify whether early edema attenuation contributes to clinical benefit.

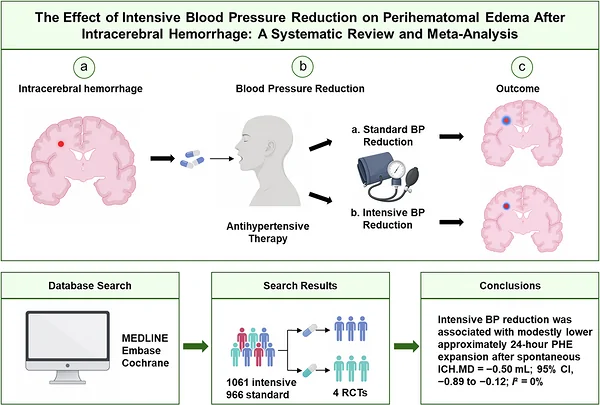

## 1 Introduction

Due to the aging of the global population, stroke and cerebrovascular diseases have become the second leading cause of death worldwide and leading causes of permanent disability; however, the treatment strategies for stroke and cerebrovascular diseases remain suboptimal (Campbell and Khatri 2020).

Hemorrhagic stroke is the second most common type of stroke and occurs due to cerebral blood vessel rupture; this type of stroke accounts for approximately 20% of all strokes and is associated with disproportionately high risks of early mortality and long‐term disability (Montano et al. 2021). Therefore, it is necessary to develop treatments that are both practical and effective to alleviate the devastating outcomes and heavy global burden of stroke and cerebrovascular diseases (Zhang et al. 2021).

Perihematomal edema (PHE), which can be detected in the low‐density zone surrounding hemorrhagic foci on computed tomography (CT), is regarded as a radiological marker of intracerebral hemorrhage (ICH)‐induced secondary brain injury (Greenberg et al. 2022). In recent years, an increasing number of studies have examined PHE as a potential therapeutic target and a surrogate measure for assessing the efficacy of emerging therapies (Wan et al. 2023).

High systolic blood pressure (SBP) may promote hematoma enlargement and exacerbate PHE after ICH (Jiang et al. 2022), and elevated blood pressure (BP) at admission is correlated with worse neurological outcomes and high rates of early and late mortality (Green and Hemphill 2022). Therefore, intensive BP reduction may be a practical therapeutic strategy in acute ICH. Many previous studies have explored the effect of intensive BP reduction on PHE, but the results have been inconsistent, and the true effect of early intensive BP reduction remains uncertain (Greenberg et al. 2022; Jiang et al. 2022).

To summarize the existing evidence and further explore the therapeutic value of early intensive BP reduction for PHE, we conducted a systematic review and meta‐analysis of studies that assessed the association between early intensive BP reduction and PHE‐related outcomes in patients with spontaneous ICH, particularly approximately 24‐h PHE expansion.

## 2 Methods

### 2.1 Search Strategy

We registered the protocol for this systematic review and meta‐analysis in the International Prospective Register of Systematic Reviews (PROSPERO; registration ID: CRD42023405336). The review was conducted and reported in accordance with the Preferred Reporting Items for Systematic Reviews and Meta‐Analyses (PRISMA) 2020 statement. We systematically searched MEDLINE via Ovid, Embase via Ovid, and the Cochrane Library from database inception to January 2026 to identify randomized controlled studies comparing intensive versus standard blood pressure reduction in patients with spontaneous ICH and reporting PHE‐related outcomes. The search strategy combined terms related to ICH, blood pressure lowering, antihypertensive treatment, and randomized or controlled trials. The full search strategies for each database are provided in Supplementary Appendix 1. The search date and final analytic approach were updated from the registered protocol according to the available evidence and extractable outcome data.

### 2.2 Study Selection

Search records were imported into EndNote X9 (Clarivate Analytics, Philadelphia, PA, USA), and duplicate records were removed. Two reviewers independently screened titles and abstracts, followed by full‐text assessment of potentially eligible reports. Discrepancies were resolved through discussion until consensus was reached. Studies were included if they met all of the following criteria: (1) enrolled patients aged 18 years or older; (2) included patients with spontaneous ICH; (3) used a randomized controlled trial design comparing intensive blood pressure reduction with standard or guideline‐based blood pressure management; and (4) reported brain edema or PHE outcomes. Studies were excluded if they met any of the following criteria: (1) non‐human studies; (2) studies not involving spontaneous ICH; (3) non‐randomized studies; (4) studies without randomized intensive and standard blood pressure reduction groups; or (5) studies without brain edema or PHE outcomes.

All eligible studies were included in the qualitative synthesis. Studies were included in the quantitative meta‐analysis only if they reported PHE change, expansion, or expansion rate where applicable, derived from baseline CT and an approximately 24‐h follow‐up CT, with follow‐up timing defined according to each original trial protocol. Sufficient data had to be available to extract or calculate the mean difference and its corresponding variance.

### 2.3 Data Extraction and Quality Assessment

Two reviewers independently extracted data from eligible studies using a standardized data extraction form. Extracted data included author, publication year, trial name, study design, sample size, patient characteristics, blood pressure treatment targets, antihypertensive agents, imaging time points, PHE measurement methods, and edema‐related outcomes. For the quantitative meta‐analysis, we extracted PHE change, expansion, or expansion rate where applicable, together with the corresponding sample size, mean, standard deviation, standard error, 95% confidence interval, or other data required to calculate the corresponding variance.

The risk of bias of included randomized controlled trials was assessed using the Cochrane risk‐of‐bias tool (Higgins et al. 2011). The assessed domains included random sequence generation, allocation concealment, blinding of participants and personnel, blinding of outcome assessment, incomplete outcome data, selective reporting, and other potential sources of bias. Each domain was judged as having low, high, or unclear risk of bias. Two reviewers performed the assessment independently, and disagreements were resolved through discussion until consensus was reached.

### 2.4 Statistical Analysis

Statistical analyses were performed using R version 4.5.2 with the meta package. The primary effect measure was the mean difference (MD) in approximately 24‐h PHE expansion between the intensive and standard blood pressure reduction groups. The MD was selected because the included studies provided continuous edema‐related outcomes that could be expressed as between‐group differences in PHE growth, change, or expansion. When standard errors or 95% confidence intervals were reported, they were converted to standard deviations or standard errors using standard formulae recommended in the Cochrane Handbook (Higgins et al. 2024).

Pooled estimates were calculated using the inverse‐variance method, which weights each study according to the precision of its effect estimate. A fixed‐effect model was used as the primary model because only a small number of studies were eligible for quantitative synthesis, making between‐study variance estimation unstable. As a sensitivity analysis, the pooled estimate was recalculated using a random‐effects model, with between‐study variance estimated using the restricted maximum‐likelihood method. Statistical heterogeneity was assessed using Cochran's *Q* test and the *I* ^2^ statistic (Higgins and Thompson 2002). Publication bias was not formally assessed because fewer than 10 studies were included in the meta‐analysis (Higgins et al. 2024).

## 3 Results

### 3.1 Search Results and Study Characteristics

**FIGURE 1:**
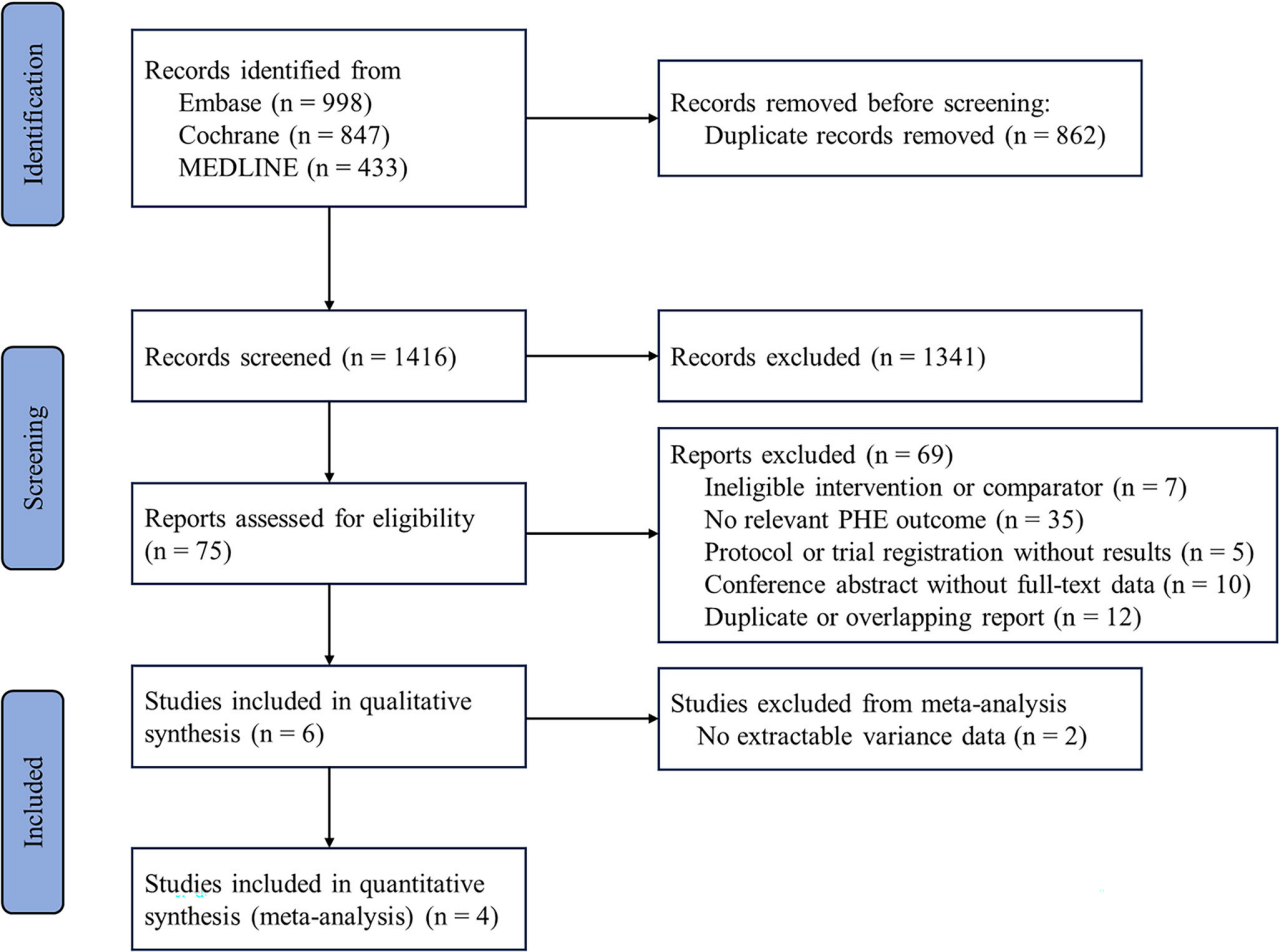
Flowchart of the study selection process.

Database searches identified 2278 records. After removal of 862 duplicates, 1416 records were screened by title and abstract, of which 1341 were excluded. The full texts of 75 reports were assessed for eligibility. A total of 6 studies met the eligibility criteria and were included in the qualitative synthesis. Among these, 4 studies provided sufficient data on approximately 24‐h PHE expansion and were included in the quantitative meta‐analysis. The 2 studies not included in the quantitative synthesis reported PHE‐related outcomes but lacked extractable variance data for the meta‐analysis. The study selection process is shown in Figure 1.

The 6 included studies were randomized trials or secondary analyses of randomized trials comparing intensive blood pressure reduction with standard or guideline‐based blood pressure management in adults with spontaneous ICH. The characteristics of these studies, including study design and hematoma location, sample size, blood pressure targets, antihypertensive agents, CT timing for PHE assessment, PHE measurement methods, and meta‐analysis status, are summarized in Table 1. The demographic and imaging characteristics of the studies included in the quantitative meta‐analysis are summarized in Tables 2 and 3, respectively.

**TABLE 1:** Study characteristics.

Data source (clinical trials)	Authors & year	Study design	Sample size	Anti‐hypertensive agents	Intensive BP target	Standard BP target	CT timing	PHE measurement method	Meta‐analysis status
	Koch et al. 2008	Single‐center feasibility RCT; supratentorial ICH	42	Labetalol, nicardipine, nitroprusside, or other agents	MAP <110 mmHg	MAP 110–130 mmHg	Baseline; 24 h post‐onset	CT; ABC/2 method; blinded assessment	Included
ICH ADAPT	McCourt et al. 2014	Secondary RCT analysis; mixed ICH locations	67	Labetalol; hydralazine; enalapril	<150 mmHg	<180 mmHg	Baseline; 2 ± 1 h; 24 ± 3 h post‐randomization	CT; Analyze 11.0; manual ROI + 5–23 HU threshold	Included
	Gong et al. 2015	RCT; basal ganglia ICH	120	IV nitroglycerin; oral ACEI after 24 h	130–140 mmHg	160–180 mmHg	Baseline; 24 h; 5 days; 14 days post‐admission	CT; ABCD/2; edema = lesion − hematoma volume	Excluded: no extractable variance data
INTERACT &INTERACT2	Yang et al. 2015	Pooled RCT CT substudy analysis; mixed ICH locations	1138	Urapidil, labetalol, hydralazine, metoprolol, CCBs, GTN/nitroprusside	<140 mmHg within 1 h	<180 mmHg	Baseline; 24 h CT	CT; MIStar v3.2; 5–33 HU threshold planimetry	Included
ATACH‐2	Leasure et al. 2019	Exploratory post hoc RCT analysis; supratentorial deep ICH	780	Intravenous nicardipine	110–139 mmHg within 2 h	140–179 mmHg within 2 h	Baseline; 24 h CT, 22.9 ± 2.6 h	CT; computerized analysis, Image‐Pro Express	Included
	Zang et al. 2019	RCT; mixed ICH locations	121	Urapidil; nifedipine after 72 h if needed	135–140 mmHg within 1 h; treatment started when SBP > 150 mmHg	<140 mmHg within 1 h; treatment started when SBP > 180 mmHg	Baseline; 24 h; 72 h; 7 days; 14 days post‐onset	CT; ABC/2 (Tada formula); blinded core assessment	Excluded: no extractable variance data

*Note*: Only 24‐h PHE data were considered for the primary analysis. Studies were excluded from the meta‐analysis when variance data for change in PHE or edema growth were not extractable.

Abbreviations: ABC/2, ellipsoid volume estimation method; ACEI, angiotensin‐converting enzyme inhibitor; ATACH‐2, Antihypertensive Treatment of Acute Cerebral Hemorrhage‐2; BP, blood pressure; CCBs, calcium channel blockers; CT, computed tomography; GTN, glyceryl trinitrate; HU, Hounsfield units; ICH ADAPT, Intracerebral Hemorrhage Acutely Decreasing Arterial Pressure Trial; INTERACT, Intensive Blood Pressure Reduction in Acute Cerebral Hemorrhage Trial; IV, intravenous; MAP, mean arterial pressure; NCCT, noncontrast computed tomography; PHE, perihematomal edema; RCT, randomized controlled trial; ROI, region of interest; SBP, systolic blood pressure.

**TABLE 2:** Demographics of included studies.

Demographics	Koch 2008	McCourt 2014 ICH ADAPT	Yang 2015 INTERACT pooled	Leasure 2019 ATACH‐2
Sample size, *n*	42	67	1138	780
Age, years, mean (SD)	60.6 (12.3)	70.1 (12.0)	65 (13)	62 (13)
Female, *n* (%)	19 (45)	18 (27)	416 (37)	289 (37)
History of hypertension (%)	37 (88)	50 (75)	819 (72)	606/755 (80)
History of ischemic stroke (%)	NR	6 (9)	103 (9)	127/776 (16)
History of antihypertensive drugs (%)	NR	31 (46)	563 (50)	361/775 (47)
Time from ICH to CT, (SD or IQR), hours	NR	2.0 (IQR 2.1)/2.6 (IQR 2.3)^b^	1.8 (1.2–2.6)	1.65 (0.85)^a^
Admission SBP, (SD), mmHg	NR	182.8 (21.3)^a^	180 (17)	175.5 (25.5)^a^
Admission DBP, (SD), mmHg	NR	95.0 (18.5)^a^	100 (15)	112.0 (20.5)^a^
GCS score	15 (10–15)/13.5 (9–15)^c^	15 (IQR 3)/15 (IQR 2)^b^	14 (13–15)	15 (13–15)

*Note*: Values are presented as mean (SD), median (IQR), median (range), or *n* (%), as indicated.

^a^Calculated by pooling arm‐specific mean and SD values.

^b^Values are shown as intensive/control because the overall value was not directly reported.

^c^Koch et al. reported GCS as median (range), not median (IQR). Koch et al. reported admission MAP, but not admission SBP or DBP; time from ICH onset to CT was not directly reported.

Abbreviations: ATACH‐2, Antihypertensive Treatment of Acute Cerebral Hemorrhage‐2; CT, computed tomography; DBP, diastolic blood pressure; GCS, Glasgow Coma Scale; ICH ADAPT, Intracerebral Hemorrhage Acutely Decreasing Arterial Pressure Trial; INTERACT, Intensive Blood Pressure Reduction in Acute Cerebral Hemorrhage Trial; IQR, interquartile range; NR, not reported; SBP, systolic blood pressure; SD, standard deviation.

**TABLE 3:** Imaging characteristics of included studies.

Imaging characteristics	Koch 2008	McCourt 2014/ICH ADAPT	Yang 2015/INTERACT pooled	Leasure 2019/ATACH‐2				
	Intensive group	Control group	Intensive group	Control group	Intensive group	Control group	Intensive group	Control group
Sample size, *n*	21	21	34	33	665^a^/601^b^	645^a^/537^b^	405	375
Baseline hematoma volume, mL	12.5 ± 17.2; SD	8.5 ± 9.8; SD	14.1 (22.2); IQR	17.2 (23.5); IQR	15.3 ± 15.4; SD^a^	14.5 ± 14.13; SD^a^	8.9 (4.8–16.4); IQR	9.4 (4.6–17.1); IQR
Baseline PHE volume, mL	26.6 ± 33.6; SD	16.3 ± 18.42; SD	0.16 ± 0.12; SD	0.15 ± 0.13; SD	2.67^b^	2.47^b^	1.4 (0.6–2.3); IQR	1.3 (0.7–2.4); IQR
24 h Hematoma volume, mL	NR	NR	14.3 (28.6); IQR	16.9 (28.5); IQR	17.4 ± 18.7; SD^a^	19.2 ± 22.7; SD^a^	9.6 (4.7–18.7); IQR	10.5 (5.3–21.2); IQR
24 h PHE volume, mL	NR	NR	NR	NR	NR	NR	1.6 (0.8–3.3); IQR	2.1 (0.9–4.1); IQR
Hematoma expansion, mL	2.4 ± 5.3; SD	2.4 ± 6.7; SD	0.74 (5.0); IQR	0.68 (4.6); IQR	NR	NR	3.0 ± 8.7; SD	4.0 ± 9.1; SD
PHE expansion, mL	7.5 ± 22.0; SD	8.6 ± 16.5; SD	0.89 (2.7); IQR	1.3 (3.4); IQR	4.77^b^ (95% CI 3.91–5.63)	5.65^b^ (95% CI 4.81–6.48)	0.9 ± 3.4; SD	1.3 ± 3.3; SD

*Note*: Values are presented as mean ± SD, median (IQR), median only, or adjusted estimate (95% CI), as indicated.

^a^ For Yang et al., hematoma volume data were calculated by pooling arm‐specific CT subgroup data from the original INTERACT and INTERACT2 trial reports; these sample sizes correspond to the hematoma imaging cohort (Anderson et al. 2008; Anderson et al. 2013).

^b^ For Yang et al., PHE data were extracted from the Yang et al. 2015/INTERACT pooled PHE analysis; these sample sizes correspond to the PHE analysis cohort used for the PHE growth comparison and quantitative meta‐analysis. The PHE analysis cohort differed from the hematoma imaging cohort. For Yang et al., baseline PHE values are reported medians, whereas PHE growth/expansion values are adjusted estimates, not raw mean ± SD.

Abbreviations: ATACH‐2, Antihypertensive Treatment of Acute Cerebral Hemorrhage‐2; CI, confidence interval; CT, computed tomography; ICH ADAPT, Intracerebral Hemorrhage Acutely Decreasing Arterial Pressure Trial; INTERACT, Intensive Blood Pressure Reduction in Acute Cerebral Hemorrhage Trial; IQR, interquartile range; NR, not reported; PHE, perihematomal edema; SD, standard deviation.

### 3.2 Quality Assessment

**FIGURE 2:**
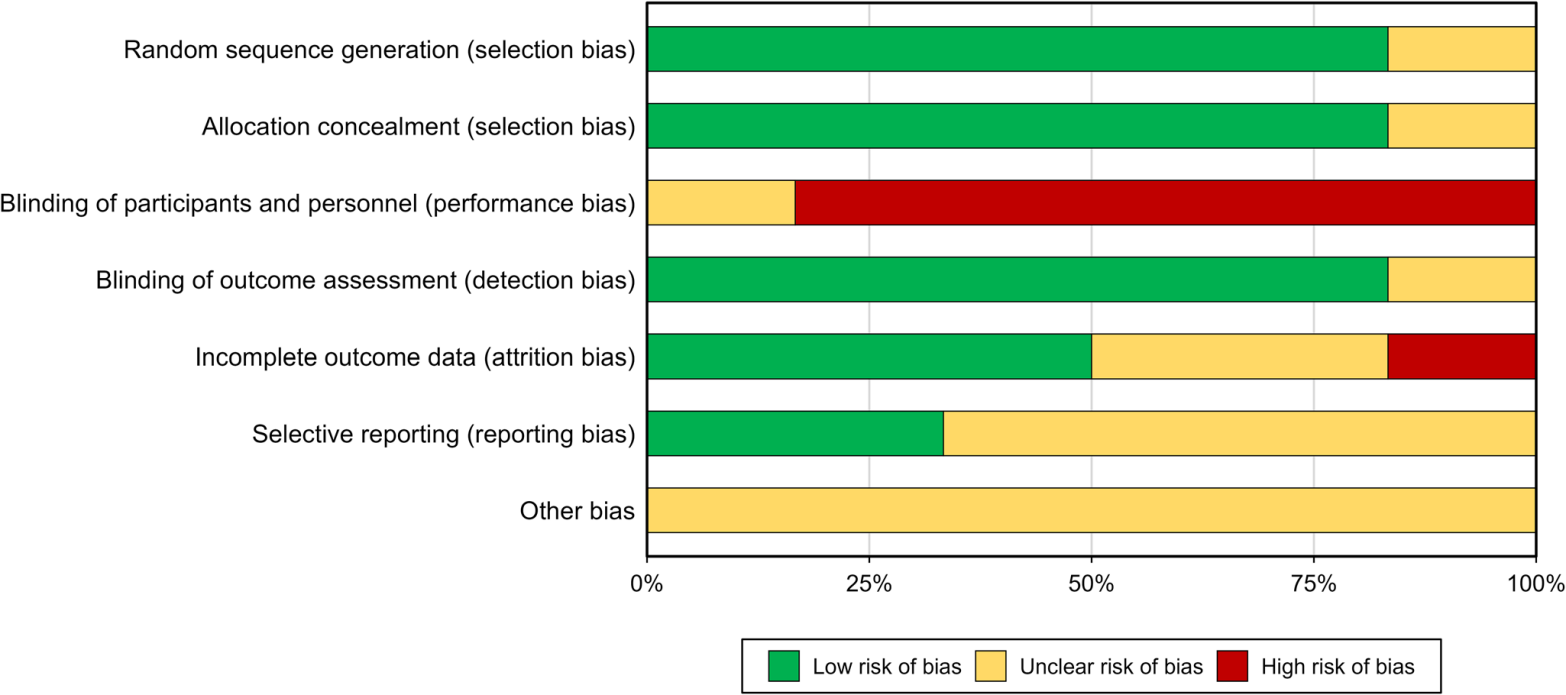
Quality assessment.

All 6 included studies employed randomized controlled designs or were secondary analyses derived from randomized trials. Koch et al. used numbered envelopes for treatment allocation, and neurological and radiological outcomes were assessed blinded to treatment allocation (Koch et al. 2008). The ATACH‐2 trial used web‐based randomization; treating physicians were not blinded because of the nature of BP management, but clinical outcomes and CT‐based ICH/PHE measurements were assessed by blinded personnel (Qureshi and Palesch 2011). The INTERACT and INTERACT2 trials used secure web‐based randomization systems with open‐label BP management; however, the risk of bias was minimized by documenting post‐randomization treatments and by assessing imaging measurements and clinical outcomes in a standardized and blinded manner (Anderson et al. 2008; Anderson et al. 2013). The ICH ADAPT protocol specified allocation using a random number generator before trial commencement, with treatment assignments stored in sequentially opened sealed, opaque envelopes at each site to assure allocation concealment (Butcher et al. 2010). Gong et al. (2015) reported random allocation to strengthened versus standard antihypertensive treatment, but details of allocation concealment and blinding were limited. Zang et al. (2019) used SAS‐generated random sequences and sealed envelopes, and reported blinded outcome assessment. The main potential source of bias was the lack of blinding of treating clinicians because BP‐target interventions were generally open‐label, whereas blinded or independent imaging assessment reduced detection bias for PHE outcomes. The detailed results of the risk‐of‐bias assessment using the Cochrane risk‐of‐bias tool are shown in Figure 2.

### 3.3 Qualitative Synthesis

There were 6 studies involving BP management and PHE. The findings were not fully consistent, reflecting differences in BP targets, imaging timing, edema definitions, and analytic approaches. Koch et al. found no significant difference in hematoma or edema growth between standard and aggressive BP reduction, although the direction of the edema estimate favored aggressive BP lowering (Koch et al. 2008). McCourt et al. (2014), using ICH ADAPT data, measured edema with manual regions of interest (ROIs) and a 5–23 Hounsfield unit (HU) threshold method and found no significant difference in absolute or relative edema growth between lower and higher SBP targets during the first 24 h; neither perihematoma cerebral blood flow nor SBP reduction predicted edema growth. Yang et al. analyzed pooled INTERACT and INTERACT2 CT substudy data and reported smaller adjusted 24‐h PHE growth in the intensive BP reduction group than in the guideline group; PHE growth was also independently associated with 90‐day death or dependency. However, randomized intensive BP treatment was not retained as an independent predictor of PHE growth in the multivariable predictor model (Yang et al. 2015). Leasure et al. (2019) specifically evaluated perihematomal edema expansion rate (PHER) in deep ICH and found that intensive BP reduction was associated with decreased 24‐h PHER, with a stronger clinically relevant signal in basal ganglia hemorrhage than in thalamic hemorrhage.

Gong et al. (2015) and Zang et al. (2019) reported findings favoring strengthened or early intensive antihypertensive treatment, particularly at later imaging time points, but these 2 studies did not provide extractable variance data for 24‐h PHE change or edema growth and were retained in the qualitative synthesis only. Taken together, the qualitative evidence suggests that intensive BP reduction may attenuate PHE expansion in selected settings, especially when edema is assessed as PHER or at later follow‐up time points. The inconsistent findings likely reflect differences in PHE measurement methods, follow‐up CT timing, hematoma location, achieved BP separation, sample size, and availability of extractable variance data.

### 3.4 Quantitative Meta‐Analysis

**FIGURE 3:**
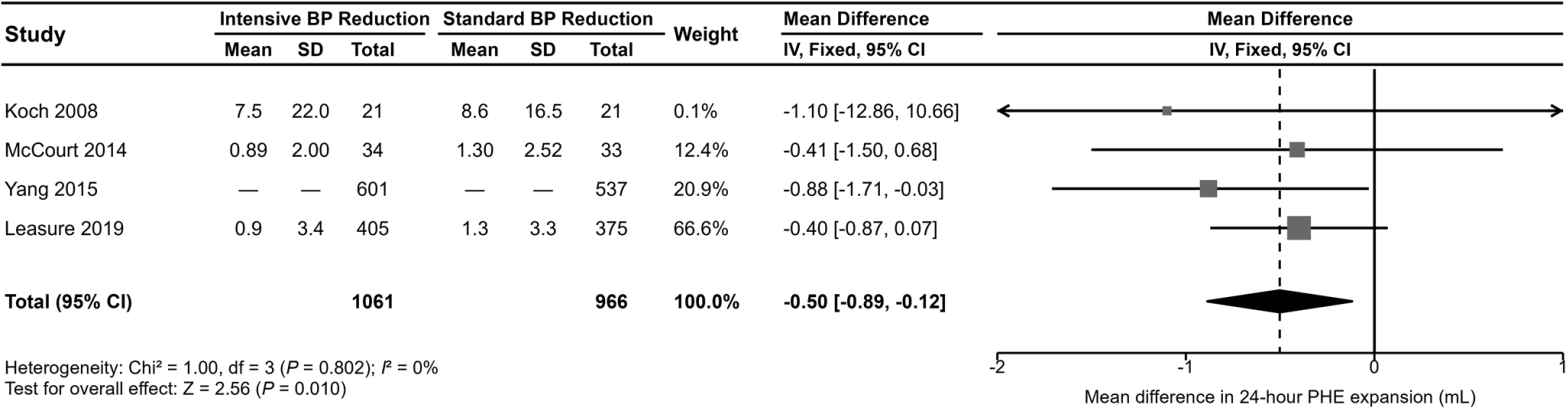
Forest plot.

The quantitative meta‐analysis included 4 studies. Intensive BP reduction was associated with smaller approximately 24‐h PHE change or expansion than standard BP management (MD = −0.50 mL; 95% CI, −0.89 to −0.12; *p* = 0.010). Yang et al. reached statistical significance, while the other 3 studies showed consistent but nonsignificant effects favoring intensive BP reduction. No statistical heterogeneity was observed (χ^2^ = 1.00, df = 3, *p* = 0.802; *I* ^2^ = 0%). The pooled estimate was unchanged in a sensitivity analysis using a random‐effects model (MD = −0.50 mL; 95% CI, −0.89 to −0.12; *p* = 0.010). Figure 3 presents the primary fixed‐effect analysis.

## 4 Discussion

This systematic review and meta‐analysis suggests that intensive BP reduction may attenuate early PHE expansion after spontaneous ICH. The pooled result favored intensive BP reduction, and the direction of effect was consistent across the quantitatively synthesized studies. These findings support a potential role of BP control in modulating early edema formation after ICH.

PHE is an important component of secondary brain injury after ICH and may aggravate mass effect, increase intracranial pressure (ICP), and worsen neurological outcomes (Marchina et al. 2022). Early PHE formation is related to hematoma‐driven processes, including clot retraction, thrombin activation, inflammatory responses, erythrocyte lysis, blood–brain barrier disruption, and hydrostatic or osmotic pressure gradients (Bautista et al. 2021; Chen et al. 2021). Because BP may influence hematoma expansion and hydrostatic pressure around the hematoma, it is biologically plausible that early BP reduction could attenuate early PHE formation (Chen et al. 2021; Minhas et al. 2022).

This meta‐analysis indicated that intensive BP reduction was associated with smaller approximately 24‐h PHE expansion. BBB disruption after ICH permits the movement of water and plasma proteins into the perihematomal region, and the hydrostatic pressure gradient, which includes ICP and BP, contributes to subsequent vasogenic PHE formation (Jiang et al. 2022; Stokum et al. 2016). Therefore, uncontrolled BP may exacerbate early PHE formation by increasing hydrostatic pressure. However, BP reduction after ICH requires attention to both degree and timing, because excessive BP reduction may compromise cerebral perfusion and very early vasodilatory treatment may interfere with early hemostatic processes (Bath et al. 2019; Stokum et al. 2016). Bath et al. (2019) reported that glyceryl trinitrate (GTN), a nitric oxide donor administered within 2 h of ICH onset, was associated with increased PHE on day 2 after ICH onset, underscoring the importance of treatment timing.

The absolute values of approximately 24‐h PHE expansion varied substantially across the 4 studies included in the meta‐analysis. This variation likely reflects differences in baseline hematoma and PHE volumes, hematoma location, analysis population, CT‐based measurement methods, imaging protocols, and analytic approaches. For example, Koch et al. (2008) reported substantially larger baseline PHE volumes and wider variability than the other studies, whereas McCourt et al. (2014) reported very small edema volumes using manual regions of interest combined with a 5–23 Hounsfield unit threshold. Yang et al. (2015) reported larger absolute PHE growth than McCourt et al. (2014) and Leasure et al. (2019), but the Yang estimates were adjusted PHE growth values derived from the INTERACT pooled PHE analysis rather than raw arm‐level mean ± SD data (Yang et al. 2015). Leasure et al. (2019) analyzed deep ICH from ATACH‐2 and reported smaller baseline hematoma and PHE volumes than the INTERACT pooled cohort. These differences indicate that the absolute magnitude of PHE expansion should be interpreted in the context of each study's imaging protocol and analysis population. Nevertheless, all 4 study‐level estimates favored intensive BP reduction, and no statistical heterogeneity was observed in the pooled analysis.

Different PHE metrics may capture different aspects of edema evolution and may therefore show different associations with BP reduction. Absolute PHE growth reflects volume change in milliliters, whereas relative PHE growth depends on baseline edema volume and may be more sensitive to small baseline values. PHER relates edema expansion to hematoma volume and may better reflect edema formation relative to hematoma burden, particularly in deep ICH (Selim and Norton 2020). Thus, intensive BP reduction could theoretically be associated with different effect sizes across these metrics, even if the direction of effect is similar. The type of PHE measure may partly account for the differing mean PHE expansion values across studies, but it is unlikely to be the sole explanation. Other contributing factors may include baseline hematoma and PHE volumes, hematoma location, CT‐based measurement methods, imaging protocols, analysis populations, and the use of adjusted estimates rather than raw arm‐level mean ± SD data (Koch et al. 2008; McCourt et al. 2014; Leasure et al. 2019; Yang et al. 2015). Because the included studies did not consistently report multiple PHE metrics and individual participant data were unavailable, we could not formally compare treatment effects across absolute PHE growth, relative PHE growth, and PHER. Future studies should prespecify standardized PHE metrics and report both absolute and normalized edema measures.

There are several limitations of this meta‐analysis. First, only 6 eligible studies were identified, and only 4 provided sufficient data for quantitative synthesis, which limited the statistical power and the precision of between‐study heterogeneity estimation. Although the random‐effects sensitivity analysis yielded an identical pooled estimate, the result should be interpreted primarily as a summary of the included studies rather than as a broadly generalizable population‐level effect. Second, the analysis was based on published summary data rather than individual participant data, which precluded subgroup analyses according to hematoma location, baseline hematoma volume, achieved BP, and imaging timing. Subgroup meta‐analysis according to hematoma location was not feasible because location‐specific PHE effect estimates were not consistently available across the included studies. Third, PHE measurement methods, imaging time points, and analytic approaches differed across studies, and the analysis focused mainly on approximately 24‐h PHE expansion rather than later or peak PHE evolution. Some data required conversion from reported confidence intervals. In addition, Yang et al. contributed adjusted PHE growth estimates, whereas the remaining studies predominantly contributed estimates derived from raw arm‐level data. Pooling adjusted and unadjusted estimates may have introduced methodological heterogeneity because of differences in covariate adjustment and effect estimation. Finally, publication bias could not be formally assessed because fewer than 10 studies were included in the meta‐analysis.

Previous trials of intensive BP reduction in ICH have reported inconsistent effects on clinical outcomes. More recent evidence from INTERACT3 and related bundled‐care initiatives suggests that BP control may be more clinically relevant when implemented as part of rapid, protocolized acute ICH management (Li et al. 2024; Ma et al. 2023; Parry‐Jones et al. 2024). The modest reduction in early PHE expansion observed in this meta‐analysis may provide an imaging‐based explanation for one potential effect of timely BP control, but it does not establish BP lowering alone as an edema‐targeted treatment sufficient to improve clinical outcomes. Future studies should determine whether standardized early BP control reduces PHE across prespecified imaging intervals and whether this imaging effect contributes to functional benefit.

## 5 Conclusions

In conclusion, intensive BP reduction was associated with a modest reduction in early, approximately 24‐h PHE expansion after spontaneous ICH. These findings suggest that early BP control may be associated with attenuation of early edema evolution; however, the limited number of eligible studies, differences in PHE measurement, and lack of individual participant data preclude firm conclusions. The results should not be interpreted as evidence that BP lowering alone is an established edema‐targeted therapy. The main take‐home message is that PHE should be considered in future trials of acute BP management after ICH, with standardized imaging protocols and prespecified edema metrics to determine whether attenuation of PHE contributes to clinical benefit.

## Author Contributions

Conception: **Xianxian Li**, **Chengguang Zou**, **Shanshan Huang**, and **Suiqiang Zhu**. Literature search: Xianxian Li and Chengguang Zou. Design: Xianxian Li, Chengguang Zou, **Yuchao Jia**, **Chensheng Pan**, and **Xiaodong Ye**. Data analysis and writing: Xianxian Li and Chengguang Zou. Funding acquisition: Yuchao Jia, Xiaodong Ye, and Shanshan Huang. Approval of final draft: all authors.

## Funding

This work was supported by the Noncommunicable Chronic Diseases‐National Science and Technology Major Project (Grant No. 2023ZD0515600; grant recipient: Yuchao Jia), the National Natural Science Foundation of China (Grant No. 82401567; grant recipient: Xiaodong Ye), the Natural Science Foundation of Hubei Province (Grant No. 2025AFB499; grant recipient: Shanshan Huang), and the Science Foundation of Tongji Hospital (Grant No. 2024A24; grant recipient: Shanshan Huang). The funders had no role in the conduct of the study or preparation of the manuscript.

## Ethics Statement

This study was a systematic review and meta‐analysis of previously published studies and did not involve direct contact with human participants or access to individual identifiable data. Therefore, ethical approval and informed consent were not required.

## Conflicts of Interest

The authors declare that they have no conflicts of interest.

## Declarations

The manuscript complies with the journal's Instructions for Authors. All authors have read and approved the submitted version of the manuscript. The data reported in this manuscript have not been published previously and are not under consideration for publication elsewhere.

## Supporting information



The full search strategies for each database are provided in Supplementary Appendix 1.

## Data Availability

All data analyzed in this study were extracted from previously published articles.
