# Supplementary material for: The Effect of Intensive Blood Pressure Reduction on Perihematomal Edema After Intracerebral Hemorrhage: A Systematic Review and Meta‐Analysis
Source: Brain Behav. 2026 Sep 14;16(9):e71724. doi: 10.1002/brb3.71724 (PMC13575631; doi:10.1002/brb3.71724)
Supplement: Supplementary file 1 [file BRB3-16-e71724-s001.docx]

**Supplementary Appendix 1. Full search strategies**

Search strategies are presented as database-specific text to preserve the original syntax, line numbering, and hit counts from each platform.

**Embase**

Platform: Ovid. Coverage: 1974 to January 21, 2026. Final yield: 998 records.

```
1   exp intracerebral hemorrhage/ or exp cerebral hemorrhage/ or exp intracranial hemorrhage/    212155
2   (intracerebral hemorrhag* or intracerebral haemorrhag* or intraparenchymal hemorrhag* or intraparenchymal haemorrhag* or cerebral hemorrhag* or cerebral haemorrhag* or brain hemorrhag* or brain haemorrhag* or intracranial hemorrhag* or intracranial haemorrhag* or hemorrhagic stroke or haemorrhagic stroke or ICH).ti,ab,kw.    112084
3   (intracerebral hematoma* or intracerebral haematoma* or intraparenchymal hematoma* or intraparenchymal haematoma*).ti,ab,kw.    3638
4   1 or 2 or 3    245118
5   ((blood pressure or BP or systolic or "systolic blood pressure" or diastolic) adj3 (lower* or reduc* or control* or manage* or treat* or target* or goal*) adj3 (intens* or aggressive or strict or tight or rapid or early or immediate)).ti,ab,kw.    7119
6   ("blood pressure lowering" or "blood pressure reduction" or "blood pressure management" or "intensive blood pressure" or "intensive blood pressure lowering" or "intensive blood pressure reduction").ti,ab,kw.    16349
7   (INTERACT or INTERACT2 or "INTERACT 2" or ATACH or ATACH2 or "ATACH-2" or "ICH-ADAPT" or "ICH ADAPT").ti,ab,kw.    245775
8   5 or 6 or 7    266644
9   randomized controlled trial/ or randomization/ or double blind procedure/ or single blind procedure/ or crossover procedure/ or placebo/    1592366
10  (randomi?ed or randomly or trial or placebo or "controlled trial").ti,ab,kw.    2889560
11  9 or 10    3210586
12  4 and 8 and 11    998
```

**MEDLINE**

Platform: Ovid. Coverage: 1946 to January 22, 2026. Final yield: 433 records.

```
1   exp Intracerebral Hemorrhage/ or exp Cerebral Hemorrhage/ or exp Intracranial Hemorrhages/    86410
2   (intracerebral hemorrhag* or intracerebral haemorrhag* or intraparenchymal hemorrhag* or intraparenchymal haemorrhag* or cerebral hemorrhag* or cerebral haemorrhag* or brain hemorrhag* or brain haemorrhag* or intracranial hemorrhag* or intracranial haemorrhag* or hemorrhagic stroke or haemorrhagic stroke or ICH).ti,ab,kw.    67743
3   (intracerebral hematoma* or intracerebral haematoma* or intraparenchymal hematoma* or intraparenchymal haematoma*).ti,ab,kw.    2980
4   1 or 2 or 3    126174
5   ((blood pressure or BP or systolic or "systolic blood pressure" or diastolic) adj3 (lower* or reduc* or control* or manage* or treat* or target* or goal*) adj3 (intens* or aggressive or strict or tight or rapid or early or immediate)).ti,ab,kw.    4214
6   ("blood pressure lowering" or "blood pressure reduction" or "blood pressure management" or "intensive blood pressure" or "intensive blood pressure lowering" or "intensive blood pressure reduction").ti,ab,kw.    10912
7   (INTERACT or INTERACT2 or "INTERACT 2" or ATACH or ATACH2 or "ATACH-2" or "ICH-ADAPT" or "ICH ADAPT").ti,ab,kw.    209658
8   5 or 6 or 7    223257
9   randomized controlled trial.pt.    653285
10  controlled clinical trial.pt.    95754
11  randomi?ed.ab.    867901
12  placebo.ab.    265928
13  randomly.ab.    478684
14  trial.ab.    797549
15  groups.ab.    2978674
16  9 or 10 or 11 or 12 or 13 or 14 or 15    4283236
17  exp animals/ not humans.sh.    5417294
18  16 not 17    3733540
19  4 and 8 and 18    433
```

**Cochrane Library**

Date searched: January 23, 2026. The final search line identified 854 hits; after excluding 7 Cochrane Review records, 847 trial records were exported for screening.

```
#1   MeSH descriptor: [Cerebral Hemorrhage] explode all trees    1658
#2   MeSH descriptor: [Intracranial Hemorrhages] explode all trees    3334
#3   (intracerebral hemorrhag* OR intracerebral haemorrhag* OR intraparenchymal hemorrhag* OR intraparenchymal haemorrhag* OR cerebral hemorrhag* OR cerebral haemorrhag* OR brain hemorrhag* OR brain haemorrhag* OR intracranial hemorrhag* OR intracranial haemorrhag* OR hemorrhagic stroke OR haemorrhagic stroke OR ICH):ti,ab,kw    16843
#4   (intracerebral hematoma* OR intracerebral haematoma* OR intraparenchymal hematoma* OR intraparenchymal haematoma*):ti,ab,kw    1022
#5   #1 OR #2 OR #3 OR #4    17233
#6   MeSH descriptor: [Antihypertensive Agents] explode all trees    10811
#7   ((blood pressure OR BP OR systolic OR "systolic blood pressure" OR diastolic) NEAR/3 (lower* OR reduc* OR control* OR manage* OR treat* OR target* OR goal*) NEAR/3 (intens* OR aggressive OR strict OR tight OR rapid OR early OR immediate)):ti,ab,kw    2519
#8   ("blood pressure lowering" OR "blood pressure reduction" OR "blood pressure management" OR "intensive blood pressure"):ti,ab,kw    4441
#9   (INTERACT OR INTERACT2 OR "INTERACT 2" OR ATACH OR ATACH2 OR "ATACH-2" OR "ICH-ADAPT" OR "ICH ADAPT"):ti,ab,kw    4266
#10  (nicardipine OR clevidipine OR labetalol OR urapidil OR hydralazine):ti,ab,kw    2642
#11  #6 OR #7 OR #8 OR #9 OR #10    21579
#12  #5 AND #11    854
```
